# Study on the Dispersion and Processing Performance of Activated Aluminum Hydroxide/Ammonium Polyphosphate Composite Flame Retardant System for Vinyl Ester Resin

**DOI:** 10.3390/polym17050667

**Published:** 2025-02-28

**Authors:** Jipeng Dou, Yong Xie, Rui Chen, Yan Qin

**Affiliations:** School of Materials Science and Engineering, Wuhan University of Technology, Wuhan 430070, China; 330913@whut.edu.cn (J.D.); xy31137@whut.edu.cn (Y.X.); 344756@whut.edu.cn (R.C.)

**Keywords:** surface modification, vinyl resin, flame retardant, stearic acid, inorganic flame retardants

## Abstract

Stearic acid was used to modify the surface of a mixed flame-retardant powder consisting of aluminum hydroxide and ammonium polyphosphate by an uneven nucleation method, aiming to improve its dispersion in a vinyl resin matrix. This study investigated the effect of stearic acid dosage on the powder’s surface modification, characterized by infrared spectroscopy, activation degree, and laser particle size distribution. The dispersion of the modified powder in the resin matrix was evaluated by measuring the system viscosity, scanning electron microscopy (SEM) images, and bending performance. The results indicated that when the stearic acid content was 1%, the powder exhibited the best overall coating effect, with a uniform particle size distribution and an activation degree of 73.6%. After the composite material was added to the resin, the system viscosity was 923 mPa·s, and SEM images showed good dispersion of the powder in the resin matrix. The cured resin demonstrated a bending strength of 41.86 MPa. However, the flame retardancy slightly decreased, with the limiting oxygen index (LOI) dropping from 24.6% for the unmodified sample to 24.0%. When the stearic acid content exceeded 1%, the powder’s particle size increased dramatically. Although the activation degree also increased, the improvement was not significant. The addition of the powder to the resin resulted in a higher system viscosity, and the flame retardancy deteriorated sharply, with the vertical burning rating dropping from FV-1 to FV-2. Considering flame retardancy, mechanical properties, and processing performance, the composite material with 1% stearic acid demonstrated the best overall performance.

## 1. Introduction

Vinyl ester resin combines the advantages of epoxy resin and unsaturated polyester resin, such as low cost, ease of processing, rapid curing at room temperature, and good molding processing performance. It has been widely applied in fields such as aerospace, construction, healthcare, and clothing. However, vinyl ester resin is highly flammable and can ignite easily, and during combustion, it generates large amounts of toxic and harmful gasses, which pose a safety risk to humans [1,2,3]. Flame retardants are commonly used additives to enhance the flame-retardant properties of polymer materials, and these include inorganic flame retardants, phosphorus-based flame retardants, halogen-based flame retardants, etc. [4,5]. Halogen-based flame retardants have high efficiency, but they release highly toxic halogenated acids and carcinogenic substances when burned. These toxic substances persist in the environment for a long time, posing harm to both biological organisms and human health, which has led to increasing restrictions on their use [6,7].

Inorganic flame retardants (such as metal hydroxides, phosphates, silicates, borates, etc.) are typically derived from natural minerals, are low cost, and have low environmental impact and toxicity, meeting the requirements of sustainable development [8,9,10]. Qiu Tian [11] and others studied the flame retardant and mechanical properties of an unsaturated polyester resin–aluminum hydroxide (ATH) system. The study showed that when the mass fraction of ATH was 40 wt %, the resin system achieved the maximum bending strength and reached a V-0 flame-retardant rating. However, the LOI value at this point was only 24.6%. When the ATH mass fraction was increased to 60 wt %, the limiting oxygen index (LOI) value increased to 34.3%, significantly improving the flame retardancy. Lin [12] and others investigated the modification of vinyl resin with ammonium polyphosphate (APP). The results showed that when the mass ratio of APP to resin was 3:7, the limiting oxygen index (LOI) of the system increased from 19.4% to 30.3%, and the peak heat release rate (PHRR) during combustion decreased from 895 kW/m^2^ to 532 kW/m^2^. In thermogravimetric analysis (TGA), the residual mass improved from 2.3% to 32.1%, demonstrating excellent flame retardancy. These findings confirm that APP is a highly effective flame-retardant filler for vinyl resin. However, the poor interfacial adhesion between inorganic powders and polymers leads to poor dispersion, reduced mechanical properties, and sedimentation issues, which affect the performance of composite materials. Therefore, the surface modification of inorganic powders is an effective approach to balance the mechanical and processing performance of halogen-free flame-retardant composites [13,14,15,16].

Stearic acid is an anionic surfactant and has been widely used as a surface modifier without a coupling structure [17,18]. Many studies on stearic acid as a surface modifier indicate that it can effectively treat inorganic powders such as CaCO_3_ and Mg(OH)_2_ [19,20,21]. Huang [20] and others explored the modification mechanism of stearic acid on Mg(OH)_2_ and its effect on the properties of vinyl acetate/Mg(OH)_2_ composites. The results showed that as the stearic acid coating amount increased, the tensile strength of the composites decreased, the elongation at break increased, and the flame retardancy deteriorated. However, stearic acid surface treatment benefited the processing ability of the composites. Ma [21] and others, in order to improve the dispersion of nano-calcium carbonate in the matrix, modified the surface of nano-calcium carbonate with stearic acid. By changing the amount of stearic acid, reaction temperature, reaction time, and slurry concentration, they measured the activation and oil absorption values of nano-calcium carbonate before and after the reaction, determined the optimal reaction conditions, and observed its dispersion state in polypropylene. The results showed that the stearic acid-treated nano-calcium carbonate reduced aggregation in the matrix and helped improve its dispersion. Liu [22] employed a hydrothermal method to modify the surface of ATH. The results showed that the highest activation degree was achieved under the following conditions: a water bath temperature of 90 °C, a stearic acid dosage of 3%, and a modification time of 30 min. Infrared spectroscopy (IR) analysis confirmed the presence of chemical bonds between stearic acid and aluminum hydroxide, indicating chemical adsorption.

In this study, aluminum hydroxide (ATH) and ammonium polyphosphate (APP) were selected as flame-retardant fillers for vinyl ester resin, considering both cost and flame-retardancy performance. To improve the compatibility between inorganic flame retardants and the polymer matrix and to enhance the mechanical properties of the polymer, stearic acid was chosen as the surface modifier for the inorganic flame retardants. Using an uneven nucleation method, stearic acid-coated inorganic flame retardants were prepared, and the changes in the properties of the vinyl ester resin before and after coating were investigated.

## 2. Experimental Section

### 2.1. Materials

The materials used in this study include epoxy vinyl ester resin with a solid content of 60% (Huachang Polymer Co., Ltd., Shanghai, China), methyl ethyl ketone peroxide used as a curing agent for the resin (Wuhan Jixin Yibang Biotechnology Co., Ltd., Wuhan, China), cobalt zincate (CoZnO_2_) used as a curing accelerator for the resin (Shanghai Kaysar Chemical Co., Ltd., Shanghai, China), and stearic acid, xylene, ammonium polyphosphate, and aluminum hydroxide (Shanghai Aladdin Biochemical Technology Co., Ltd., Shanghai, China).

### 2.2. Surface Treatment of Flame-Retardant Powders

**Figure 1:**
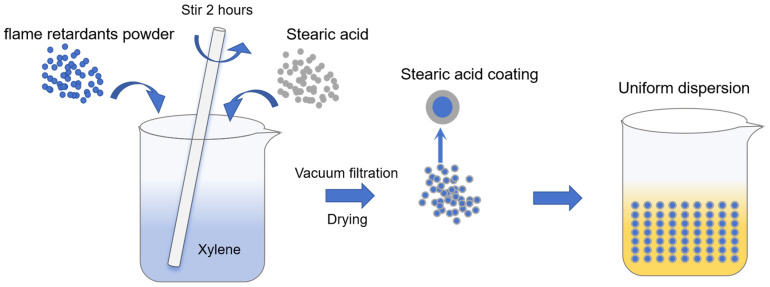
Schematic diagram of surface modification of flame-retardant powder.

To prepare the flame-retardant powder, 40 g of aluminum hydroxide (ATH) and 60 g of ammonium polyphosphate (APP) were mixed together. The resulting mixture was placed in an oven at 110 °C for 12 h to thoroughly remove the moisture, ensuring the surface of the powder was fully exposed. Afterward, 100 g of xylene was added to the powder, and the emulsion was poured into a three-neck flask equipped with a stirring mechanism. Then, according to the proportions shown in Table 1, stearic acid was added in varying mass fractions. The mixture was mechanically stirred for 2 h at a stirring speed of approximately 400 rpm. Finally, the flame-retardant powders SAA-0, SAA-1, SAA-2, SAA-3, and SAA-4 were obtained by vacuum filtration, drying, and grinding. The schematic diagram of the modification is shown in Figure 1.

### 2.3. Preparation of Flame Retardant Vinyl Ester Resin

The prepared flame-retardant powder was added to the vinyl resin according to Table 2, and the mixtures were labeled as VER-0, VER-1, VER-2, VER-3, and VER-4, respectively. The mixture was then subjected to ultrasonic stirring for 30 min to ensure thorough dispersion. Afterward, 1.2 g of cobalt zincate was added and mixed evenly, followed by the addition of 2.4 g of methyl ethyl ketone peroxide. The mixture was then poured into molds and allowed to cure at room temperature. The mechanical and flame-retardant properties of the cured resin were subsequently tested.

### 2.4. Measurement of Activation Degree

To determine the activation degree of the inorganic powder, 5.0 g of the powder was placed in a beaker, and 200 mL of deionized water was added. The mixture was stirred for 1 h and then allowed to stand. Afterward, the floating inorganic powder was removed, filtered to eliminate water, and dried in an 80 °C oven until constant weight was achieved. The activation degree (H) was calculated using the following formula:

(1) $$H=m_{float}/m_{total}\times 100\%$$

where $m_{float}$ is the mass of the floating inorganic powder (g), and $m_{total}$ is the total mass of the powder sample (g).

### 2.5. Flame-Retardancy Testing

Two standard test methods were used to evaluate the flame retardancy of the composite materials:

Limiting oxygen index (LOI) is the minimum oxygen concentration required to sustain combustion in a mixture of oxygen and nitrogen. The test was performed by vertically mounting the sample and igniting it from the top. The sample was prepared according to the national standard GB/T 2406-1993 [23] and tested using the JF-3 instrument produced by Jiangsu Province, Jiangning Analytical Instrument Factory, Nanjing, China.

The vertical burning test involves maintaining the sample in a vertical position, with the bottom exposed to the flame. The sample is prepared according to the national standard GB/T 2408-1996 [24]. Based on the time taken for combustion to stop after two 10 s applications of the flame and whether any flame dripping occurs, the material is classified into four grades: FV-0, FV-1, FV-2, and FV-3. If the flame is applied twice to the test sample, each for 10 s, and combustion stops within 10 s without flame dripping, it is classified as FV-0. If the flame stops within 30 s after two 10 s applications and there is no flame dripping, it is classified as FV-1. However, if flame dripping occurs, the composite material is classified as FV-2. If the combustion continues for more than 30 s after two 10 s flame applications, the material is classified as “-” grade. FV-0 represents the best rating, while “-” represents the worst rating. At least five specimens are tested for each experiment.

### 2.6. Other Tests and Characterization

Fourier-transform infrared (FTIR) spectroscopy was used to analyze the surface modification of the inorganic powders by stearic acid. The FTIR spectra were obtained using a Nexus FTIR spectrometer (Thermo Nicolet, Madison, WI, USA). Laser particle size distribution was analyzed using a Mastersizer 2000 laser diffraction particle size analyzer (Malvern Instruments Ltd., Malvern, UK) to evaluate the particle size, distribution, and agglomeration of the powders before and after surface modification. The viscosity of the flame-retardant-modified vinyl ester resin was measured using a rotational viscometer (NDJ-8spro, Shangyi, Shanghai, China) to study the effect of different stearic acid coatings on the resin’s processing performance. Bending strength was determined using a universal testing machine (RGM4100, Rigel, Shenzhen, China) with a fixed loading speed of 2 mm/s, according to GB/T 1449-2005 standards [25]. The dispersion of the inorganic flame-retardant powders in the vinyl ester resin was analyzed using a field emission scanning electron microscope (FE-SEM, Carl Zeiss, Oberkochen, Germany).

## 3. Results and Discussion

### 3.1. Surface Modification of Inorganic Powders by Stearic Acid

**Figure 2:**
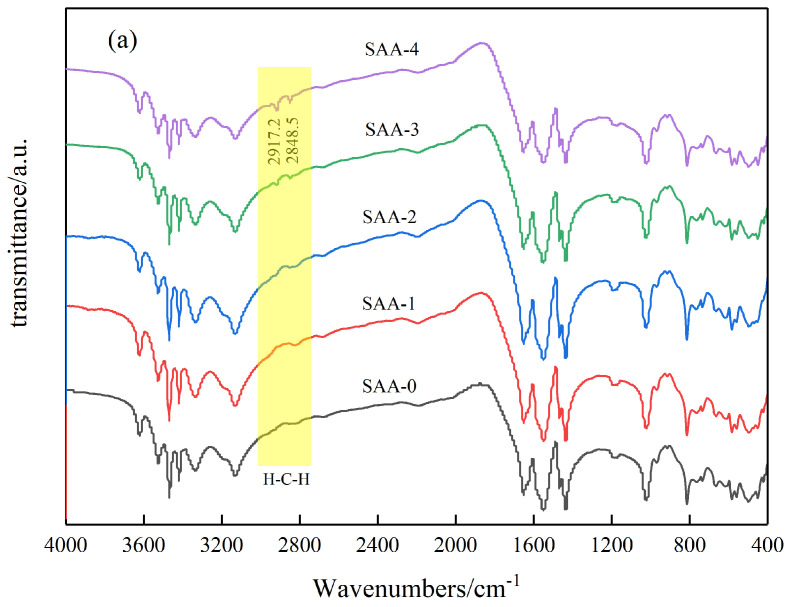

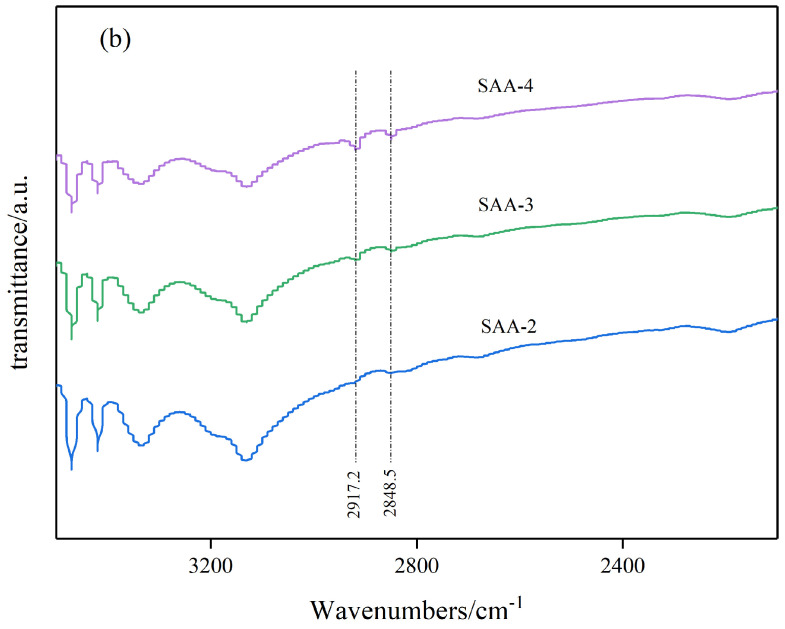
(**a**) Infrared spectra of all flame-retardant powders. (**b**) Enlarged view of the methylene peak.

Figure 2a shows the infrared spectra of the inorganic flame-retardant powders after surface modification with different amounts of stearic acid. As can be seen, the peaks at 2917.2 cm^−1^ and 2848.5 cm^−1^, which represent the asymmetric and symmetric stretching vibrations of the methylene group (H-C-H), appear in the samples SAA-2, SAA-3, and SAA-4. These peaks are more pronounced in SAA-3 and SAA-4, less noticeable in SAA-2, and absent in SAA-1 and SAA-0. Figure 2b displays a magnified view of the methylene peaks for SAA-2, SAA-3, and SAA-4. This indicates that when the content of stearic acid exceeds 1%, a strong chemical bond is formed between the stearic acid and the flame-retardant powders, covering the surface of the powders [13]. Moreover, the intensity of the chemical bonds increases with the mass fraction of stearic acid.

**Figure 3:**
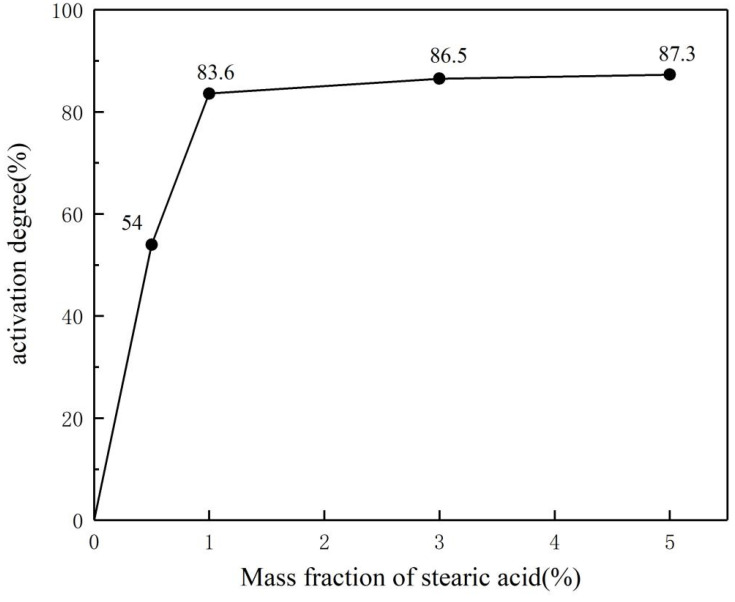
Activation degree of flame-retardant powder.

Figure 3 shows the activation degree of the inorganic flame-retardant powders after surface modification with different mass fractions of stearic acid. The activation degree reflects the extent of the surface modification of the inorganic powders. The unmodified inorganic powders have a highly polar surface, which causes them to settle in water. In contrast, the surface of the modified powders becomes non-polar and highly hydrophobic, causing them to float on the water surface, which serves as an important indicator for measuring the extent of surface modification. As shown in the figure, the activation degree of the powders increases with the mass fraction of stearic acid. However, when the mass fraction of stearic acid exceeds 1%, the increment in activation degree becomes less significant, despite the addition of a larger amount of stearic acid. This is because, at 1% mass fraction, stearic acid already forms a nearly complete monolayer coverage with the hydrophobic tail oriented outward [22]. At higher mass fractions, only multilayer stearic acid layers are formed, leading to larger particle sizes and a slight increase in hydrophobicity, but the increase is not significant.

**Figure 4:**
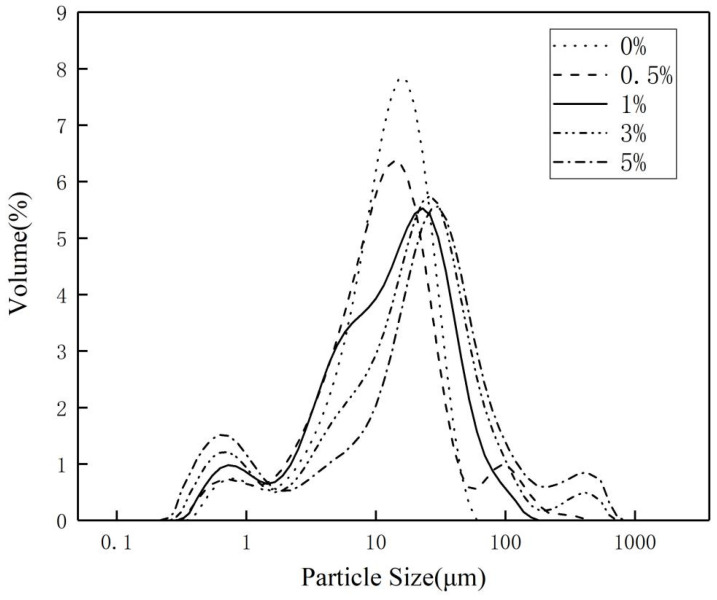
Flame-retardant powder particle size distribution map.

Figure 4 shows the laser particle size distribution of the inorganic flame-retardant powders before and after surface modification. Table 3 presents the particle size distribution data of the different flame-retardant powders. In the table, d(0.5) refers to the particle size at the 50% cumulative passing rate, commonly known as the median particle size. It indicates that half of the particles in the sample have a particle size smaller than this value, while the other half have a particle size larger than this value. d(0.5) is commonly used to describe the size characteristics of powders, particles, and granular materials. In industrial production, understanding the d(0.5) value is crucial for optimizing production processes and improving product quality. d(0.9) represents the particle size at the 90% cumulative passing rate, indicating that 90% of the particles in the sample have a particle size smaller than or equal to this value, while the remaining 10% have a particle size larger than this value. As shown in the figure, the particle size of the inorganic flame-retardant powders increases with the mass fraction of stearic acid. For SAA-4, the d(0.5) value is 97.43 µm, with the maximum particle size reaching 724.44 µm. Among all the curves, the particle size distribution of the unmodified powder is the most uniform. Compared to the surface-modified powders, the particle size distribution of the SAA-2 powder is the most even, with a d(0.5) of 13.09 µm and a maximum particle size of 138 µm. Additionally, no third peak is observed in its distribution. In the curves of SAA-1, SAA-3, and SAA-4, a third aggregation peak appears. The third peak in SAA-1 may be due to the insufficient surface modification of the inorganic flame-retardant powder by stearic acid, causing significant agglomeration during filtration and drying, which ultimately leads to larger particle sizes. The third aggregation peak in SAA-3 and SAA-4 may be due to the excessive concentration of stearic acid in the surface modification, resulting in many particles being covered by multiple layers of stearic acid.

### 3.2. Effect of Surface-Modified Inorganic Flame-Retardant Powders on the Properties of Vinyl Ester Resin

**Figure 5:**
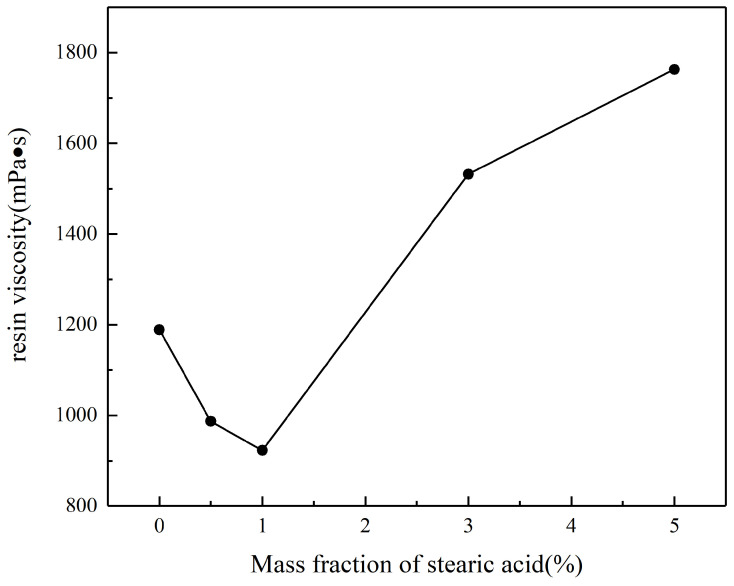
Effect of stearic acid coating on resin viscosity.

Figure 5 shows the viscosity curves of the vinyl ester resin after the addition of different flame-retardant powders. Initially, the viscosity of the resin decreased as the mass fraction of stearic acid increased. At a stearic acid mass fraction of 1%, the viscosity reached its lowest point at 923 mPa·s. However, when the stearic acid content increased to 3%, the resin viscosity sharply increased, and at a 5% stearic acid mass fraction, the viscosity reached 1763 mPa·s. This behavior is attributed to the fact that, at low concentrations, stearic acid forms a monolayer coating that is hydrophobic, which aids in the dispersion of the inorganic powders. Table 4 illustrates the relationship between viscosity and particle size distribution. It can be observed that the viscosity of the modified samples is primarily determined by the d(0.9) value. Comparing VER-1 and VER-2, when the d(0.9) values are similar, the viscosities are also comparable. In such cases, the final viscosity is influenced by the maximum particle size: the smaller the maximum particle size, the lower the viscosity [20,26]. Additionally, stearic acid has a lubricating effect, which reduces the resin’s viscosity compared to unmodified powders. However, if the stearic acid concentration is too high during surface modification, the particle size increases rapidly, leading to an overall increase in viscosity.

Table 5 shows the effect of flame-retardant powders under different modification conditions on the flame retardancy of vinyl ester resin. As the amount of stearic acid added during surface modification increases, the flame retardancy of the vinyl ester resin gradually decreases, as evidenced by the decrease in the LOI value and the reduction in the vertical burning rating. The mass fraction of stearic acid during the surface treatment of the inorganic flame-retardant powders has little effect on the LOI value of the composite materials. However, in the vertical burning test, when the mass fraction of stearic acid exceeds 3%, the burning time exceeds 30 s, and the flame-retardant rating fails to meet FV-1.

**Figure 6:**
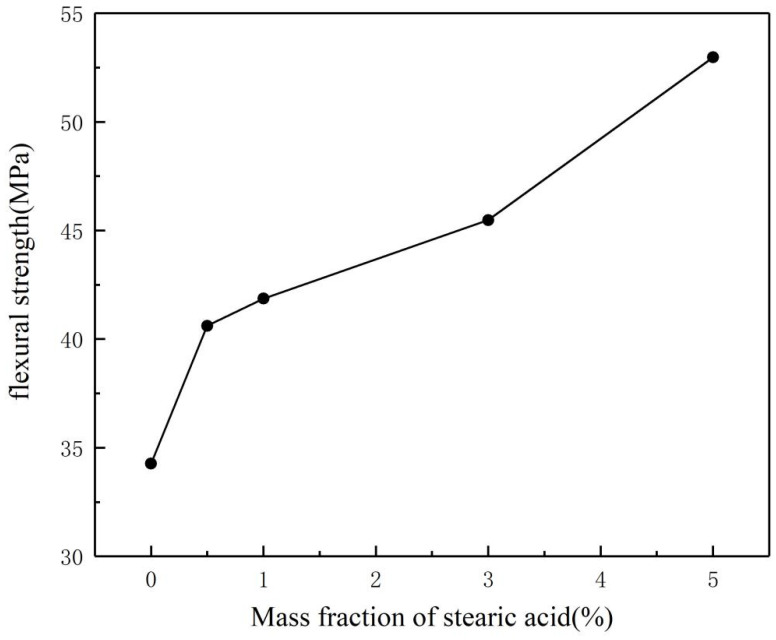
Effect of stearic acid coating on mechanical properties of composites.

Figure 6 shows the flexural strength curves of the samples. As observed, the flexural strength of the resin after curing increases with the increasing mass fraction of stearic acid, from 34.27 MPa for VER-0 to 52.97 MPa for VER-4. This improvement is due to the addition of stearic acid, which enhances the toughness of the resin matrix. The more stearic acid added, the better the toughness of the composite material. Furthermore, the surface-modified inorganic powders become more hydrophobic, which allows for uniform dispersion in the resin matrix and reduces powder agglomeration, thus preventing defects in the resin matrix. Therefore, as the mass fraction of stearic acid increases, the fracture strength of the resin also increases.

**Figure 7:**
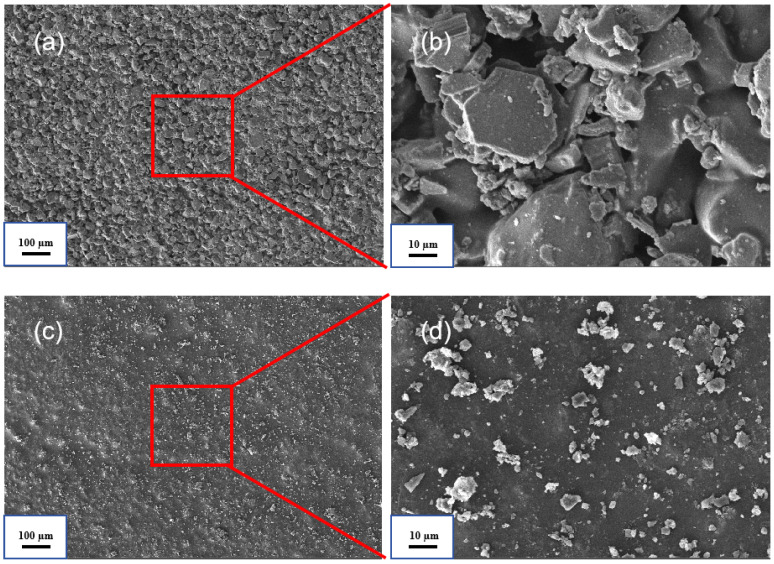
Surface topography of the composite materials. (**a**,**b**) are images of different scales of VER-0, and (**c**,**d**) are images of different scales of VER-2.

Figure 7 shows the SEM images of VER-0 and VER-2. As illustrated in Figure 7a,b, large, agglomerated flame-retardant particles with sizes around 30 µm are densely distributed. Due to the large particle size, these particles settle during resin curing, resulting in a visibly uneven surface on the composite material. In contrast, in Figure 7c,d, the powder dispersion is more uniform, and the aggregation phenomenon has improved. The maximum particle size is only 10 µm, and the surface is much smoother. This improvement is beneficial for enhancing the homogeneity of the material and its long-term storage stability. The reason for this is that the surface-modified flame-retardant powder is more hydrophobic, allowing for better dispersion in the resin matrix, which reduces the aggregation phenomenon. Moreover, the particle size distribution of SAA-2 is more uniform [20].

## 4. Conclusions

This study systematically investigates the role of stearic acid as a surface modifier for ATH/APP flame-retardant powders in vinyl ester resin composites. The following conclusions are drawn:

1. Optimal stearic acid dosage: A stearic acid content of 1 wt % achieves the best balance between particle dispersion and processing performance. The modified powder (SAA-1) exhibits uniform particle size distribution (d(0.5) = 13.09 µm), high activation degree (73.6%), and minimal resin viscosity (923 mPa·s), while maintaining a bending strength of 41.86 MPa.
2. Trade-off mechanism: Excessive SA (>1 wt %) induces particle agglomeration (d(0.5) up to 724.44 µm for SAA-4), leading to deteriorated flame retardancy (LOI decreased from 24.6% to 23.6%), despite improved mechanical strength (52.97 MPa for VER-4).
3. Industrial relevance: The proposed surface modification method demonstrates scalability potential. This study provides a cost-effective strategy for optimizing halogen-free flame-retardant composites, suitable for applications requiring both mechanical robustness and processing efficiency.
4. Industrial application potential: The proposed surface modification method demonstrates scalability potential. This modification method requires only one step, is easy to operate, and offers simplicity and effectiveness for large-scale production. This study provides an economical and efficient strategy for optimizing halogen-free flame-retardant composites.

The future research direction: Explore industrial-scale coating technologies (such as fluidized bed processes) to achieve higher particle size uniformity and a greater activation degree.

## Figures and Tables

**Table 1:** Flame-retardant powder surface treatment formulations.

Samples	ATH/G	APP/G	Stearic Acid/G
SAA-0	40	60	0
SAA-1	40	60	0.5
SAA-2	40	60	1.0
SAA-3	40	60	3.0
SAA-4	40	60	5.0

**Table 2:** Formulation of flame-retardant vinyl ester resin composites.

Samples	Resin/G	Flame-Retardant Powder Added
VER-0	120	50 g SAA-0
VER-1	120	50 g SAA-1
VER-2	120	50 g SAA-2
VER-3	120	50 g SAA-3
VER-4	120	50 g SAA-4

**Table 3:** Particle size distribution data sheet of flame-retardant powder.

Samples	d(0.5)/µm	d(0.9)/µm	Maximum Particle Size/µm
SAA-0	11.93	26.06	45.71
SAA-1	12.49	38.14	416.87
SAA-2	13.09	40.37	138.04
SAA-3	18.33	61.42	549.54
SAA-4	22.28	97.43	724.24

**Table 4:** Relationship between viscosity and particle size distribution.

Samples	d(0.9) of the Added Powder/µm	Maximum Particle Size/µm	Viscosity of the Resin/mPa·s
VER-0	26.06	45.71	1189
VER-1	38.14	416.87	987
VER-2	40.37	138.04	923
VER-3	61.42	549.54	1532
VER-4	97.43	724.24	1763

**Table 5:** Effect of stearic acid coating on flame retardancy of composites.

Samples	LOI	Vertical Fire Test
VER-0	24.6	FV-1
VER-1	24.3	FV-1
VER-2	24.0	FV-1
VER-3	23.8	FV-2
VER-4	23.6	FV-2

## Data Availability

The original contributions presented in this study are included in the article. Further inquiries can be directed to the corresponding author.
